# Medical Staff of the Queen

**Published:** 1837-10

**Authors:** 


### MEDICAL STAFF OF THE QUEEN.

The following are the medical appointments which have been made since the commencement of the present reign:—

Physicians in Ordinary:—James Clark, M.D.; Sir Henry Halford, Bart., M.D. G.C.H.; William Frederick Chambers, M.D. K.C.H.

Sergeant-Surgeons:—Sir Astley P. Cooper, Bart., G.C.H.; Sir Benjamin C. Brodie, Bart.; Robert Keate, Esq.

Physician to the Household:—James Clark, M.D.

Surgeon to the Household:—John Phillips, Esq.

Apothecary to the Person:—John Nussey, Esq., and Edward Duke Moore, Esq., jointly.

Apothecary to the Household:—John Nussey, Esq., and Charles Craddock, Esq., jointly.

Physicians Extraordinary:—Sir James M'Grigor, Bart., M.D.; Henry Holland, M.D.; Peter Mere Latham, M.D.; Richard Bright, M.D.; Neil Arnott, M.D.

Surgeons Extraordinary:—Benjamin Travers, Esq.; Thomas Copeland, Esq.; Wm. Lawrence, Esq.; Henry Earle, Esq.; Richard Blagden, Esq.

Apothecaries Extraordinary:—Messrs. Merriman, of Kensington.

